# Suicides in degenerative neurocognitive disorders and traumatic brain injuries

**DOI:** 10.1192/j.eurpsy.2024.3

**Published:** 2024-01-17

**Authors:** Tiina Talaslahti, Milena Ginters, Anniina Palm, Hannu Kautiainen, Risto Vataja, Henrik Elonheimo, Jaana Suvisaari, Hannu Koponen, Nina Lindberg

**Affiliations:** 1 Department of Psychiatry, University of Helsinki, Helsinki, Finland; 2 Primary Health Care Unit, Kuopio University Hospital, Kuopio, Finland; 3 Department of Biostatistics, Folkhälsan Research Center, Helsinki, Finland; 4 The Department of Government Services, Finnish Institute for Health and Welfare, Helsinki, Finland; 5 Mental Health Unit, Finnish Institute for Health and Welfare, Helsinki, Finland

**Keywords:** cause of death, neurocognitive disorder, register, suicide, traumatic brain injury

## Abstract

**Background:**

Neuropsychiatric symptoms in major neurocognitive disorders have been strongly associated with suicidality.

**Methods:**

The objectives were to explore suicide rates in degenerative neurocognitive disorders (DNDs), alcohol-related neurocognitive disorders (ARNDs), and traumatic brain injuries (TBIs). Patients who received these diagnoses between 1998 and 2015 (*N* = 231,817) were identified from nationwide registers, and their mortality was followed up until December 31, 2018. We calculated incidences of suicides per 100,000 person-years, types of suicides, and suicide rates compared with the general population (standardized mortality ratio [SMR]).

**Results:**

During the follow-up, 0.3% (95% confidence interval [95% CI]: 0.2–0.5) of patients with DNDs, 1.1% (0.7–1.8) with ARNDs, and 1.0% (0.7–1.3) with TBIs committed suicide. Suicide mortality rate was higher in men (58.9, 51.3, to 67.4 per 100,000) than in women (9.8, 7.5, to 12.5 per 100,000). The highest suicide rate was in ARNDs (98.8, 65.1, to 143.8 per 100,000), followed by TBIs (82.0, 62.4, to 105.8 per 100,000), and DNDs (21.2, 18.3, to 24.5 per 100,000). The SMRs (95% CI) were 3.69 (2.53–5.38), 2.99 (2.31–3.86), and 1.31 (1.13–1.51), respectively, and no sex difference emerged. The most common cause of death was self-inflicted injury by hanging or drowning (12.4, 10.3, to 14.8 per 100,000).

**Conclusions:**

Suicide rates were higher in all three patient groups than the general population. Suicide risk remained elevated for more than 10 years after diagnosis. The suicide methods were mostly violent.

## Introduction

Globally, the suicide rate of older people has increased the most relative to other age-groups [1–3]. Suicide attempts in older generations are more likely to be lethal than attempts in younger generations [4]. As the average life expectancy rises, there is also an increase in the prevalence of central nervous system (CNS) degenerative conditions associated with dementia, alcohol-related damage, and previous brain injuries. These disorders cause neuropsychiatric symptoms that can potentially elevate the risk of suicidal behavior [5–14].

Neuropsychiatric symptoms, such as depression, anxiety, and emotional/behavioral dysregulation, are strongly linked to suicidality in both degenerative neurocognitive disorders (DNDs) and traumatic brain injuries (TBIs) [10, 15–22]. The feeling of losing control and decision-making capabilities, impaired coping skills, and threats to continuity and self-identity caused by these disorders may contribute to the emergence of suicidality [23]. However, suicidal thoughts among DND patients tend to diminish as the disease progresses [20].

Studies on DND patients have shown the risk of suicide ranging from 1.5- to 3-fold [5–7]. As for TBI, the risk of suicidal behavior or death has mostly been 1.9 to 2.5 times higher than the general population [9–13]. Alcohol misuse and depression further increase the risk among individuals with DNDs and TBIs [10]. However, there is a lack of earlier literature on suicidal behavior of patients with alcohol-related neurocognitive disorders (ARNDs), that is, alcoholic Wernicke–Korsakoff syndrome (WKS) or alcohol-related dementia (ARD).

The risk period for self-harm appears to coincide when daily functioning and cognitive skills begin to deteriorate [24]. In individuals with DNDs and TBIs, the highest risk of suicide has been observed soon after receiving diagnosis, particularly among younger patients [6, 7, 25–28]. In two large studies, the risk within the first year following diagnosis has been around three-fold [6, 7, 9]. The important risk group for suicides seems to be patients younger than 65 years and within 3 months of diagnosis [28].

Based on previous literature, we hypothesized that neurocognitive disorders predispose to psychiatric symptoms and feelings of hopelessness, thus increasing the risk of suicide. Given the scarcity of data on suicide rates among individuals with DNDs, ARNDs, and TBIs, we decided to explore the suicides in these patient groups utilizing Finnish high-quality nationwide registers. Our study aims were to calculate incidences and timing of suicides related to the moment of diagnosis, cumulative incidences of suicides since diagnosis, and types of suicides, including those that involved violent methods. We also sought to examine suicide rates compared with the age- and sex-matched general population. The use of registers enabled a large study sample and long follow-up.

## Methods

### Information on diagnoses

We obtained data on patients with various diagnoses of DNDs, ARNDs, and TBIs from the Finnish Hospital Discharge Register maintained by the Finnish Institute for Health and Welfare, later known as the Finnish Care Register for Health Care (HILMO). This register contains data on inpatient care, that is, discharge from hospitals and health-care centers, since 1969, and specialized outpatient care visits since 1998, whereas the Register of Primary Health Care Visits (AvoHILMO) contains publicly funded primary health-care visits since the year 2009 [29].

Mortality statistics on the research population as well as on the age- and sex-matched general population were received from Statistics Finland. The Finnish Causes of Death register includes information on all deaths and causes of death in Finnish permanent residents and citizens since 1969 [30, 31]. Death certificates are based on the patient’s case history, and if death is unexpected or unknown, the cause of death is ascertained by a forensic autopsy. Suicide is defined as death caused by self-directed injurious behavior with an intent to die as a result of one’s behavior (codes X60–X84 and Y870) [32].

### Information on the study population

The material of our study was register-based, comprehensive, and nationwide. The sample comprised all Finnish patients who had received a diagnosis of DND, ARND, or TBI between 1998 and 2015. The patients were aged 40 years or more at diagnosis. Data on mortality were collected from registers between 1998 and 2018. The classification of disease diagnoses and causes of death was based on the 10th revision of the World Health Organization International Classification of Diseases (ICD-10) (WHO, 1992), which has been used in Finland since 1996. Data on different registers were linked by the personal identification number allocated to each Finnish citizen.

Patients with DNDs had diagnoses of Alzheimer’s disease (F00 and/or G30 codes), frontotemporal dementia (F02.0 and/or G31.0 codes), Parkinson’s disease dementia (F02.3 and/or G20 codes), dementia with Lewy bodies (F02.8 and/or G31.8 codes), vascular dementia (F01 and/or I67.3), or dementia not otherwise specified (F03) and also diagnoses of alcohol-induced amnesic syndrome, that is, WKS (F10.6), ARD (F10.73), and other chronic alcoholic brain syndrome (F10.74). In the group of head injuries, the diagnoses were sequelae of intracranial injury and organic amnesic syndrome, not induced by alcohol or other psychoactive substances (T90.5 and F04) and sequelae of intracranial injury and organic amnesic syndrome with post-concussional syndrome (T90.5 and F07.2).

Suicides were classified as follows: X60–X67 intentional self-poisoning; X70 intentional self-harm by hanging, strangulation, or suffocation; X71 intentional self-harm by drowning or submersion; X72–X78 intentional self-harm by shooting, by explosive material, burning or steaming, or by sharp object; X80 self-harm by jumping from a high place; and X81 jumping or lying in front of a moving object. Sequelae of intentional self-harm, assault, and events of undetermined intent were coded as Y87.

### Data analysis

We present descriptive statistics as means with standard deviation (SD) or as counts with percentages. Crude estimates of suicide incidence (per 100,000 person-years) or incidence rate ratios (IRRs) were calculated using Poisson regression models or random-effects negative binomial regression models (unstructured correlation structure), as appropriate. The assumptions of overdispersion in the Poisson model were tested using Lagrange multiplier test. Time-to-event evaluation was based on the product limit estimate (Kaplan–Meier estimate) of the cumulative function of the suicide rate and all-cause mortality. The ratio between observed and expected numbers, standardized mortality ratio (SMR), was calculated using subject-years methods with 95% confidence intervals (CIs), assuming Poisson distribution. The expected number of deaths was calculated on the basis of sex-, age-, and calendar-period-specific mortality rates in the Finnish population (Official Statistics of Finland). The expected number was determined by multiplying the person-years of observation with the appropriate mortality rate in the general population according to the categories of sex, 1-year age-group, and calendar period. All analyses were performed using STATA 17.0 (StataCorp LP, College Station, TX, USA).

### Ethical considerations

The study protocol was approved by the Coordinating Ethics Committee of Helsinki University Hospital (no 186/13/03/00/16). Participants are not identifiable in the register data.

## Results

### General information

In the end of the follow-up, 0.12% (*N* = 274) of the whole sample of 231,817 patients committed suicide. In the groups of DNDs, ARNDs, and TBIs, the mean ages at death were 73 (SD: 11) years, 57 (SD: 9) years, and 58 (SD: 12) years, respectively. Patients with DNDs, ARNDs, and TBIs comprised 94%, 2%, and 4% of cases, respectively. Detailed patient characteristics are presented in Table 1.

**Table 1.** General information on groups of patients with DNDs, ARNDs, and TBIs

	DND (*N* = 218,704)	ARND (*N* = 4,248)	TBI (*N* = 8,865)
Women, *n* (%)	140,515 (64)	1,034 (24)	2,440 (28)
Mean age at diagnosis, mean (SD)	80 (8)	61 (10)	58 (13)
Person-years were followed up	886,979	27,320	71,956
Number of deaths	178,966	2,450	3,033
All-cause mortality, % (95% CI)	98.6 (98.4–98.8)	83.8 (80.7–86.7)	59.9 (57.5–62.3)
All-cause SMR (95% CI)	2.43 (2.42–2.44)	5.48 (5.27–5.70)	1.96 (1.89–2.03)

Abbreviations: ARNDs, alcohol-related neurocognitive disorders; CI, confidence interval; DNDs, degenerative neurocognitive disorders; N, number; SMR, standardized mortality ratio; TBIs, traumatic brain injuries.

**Figure 1.**
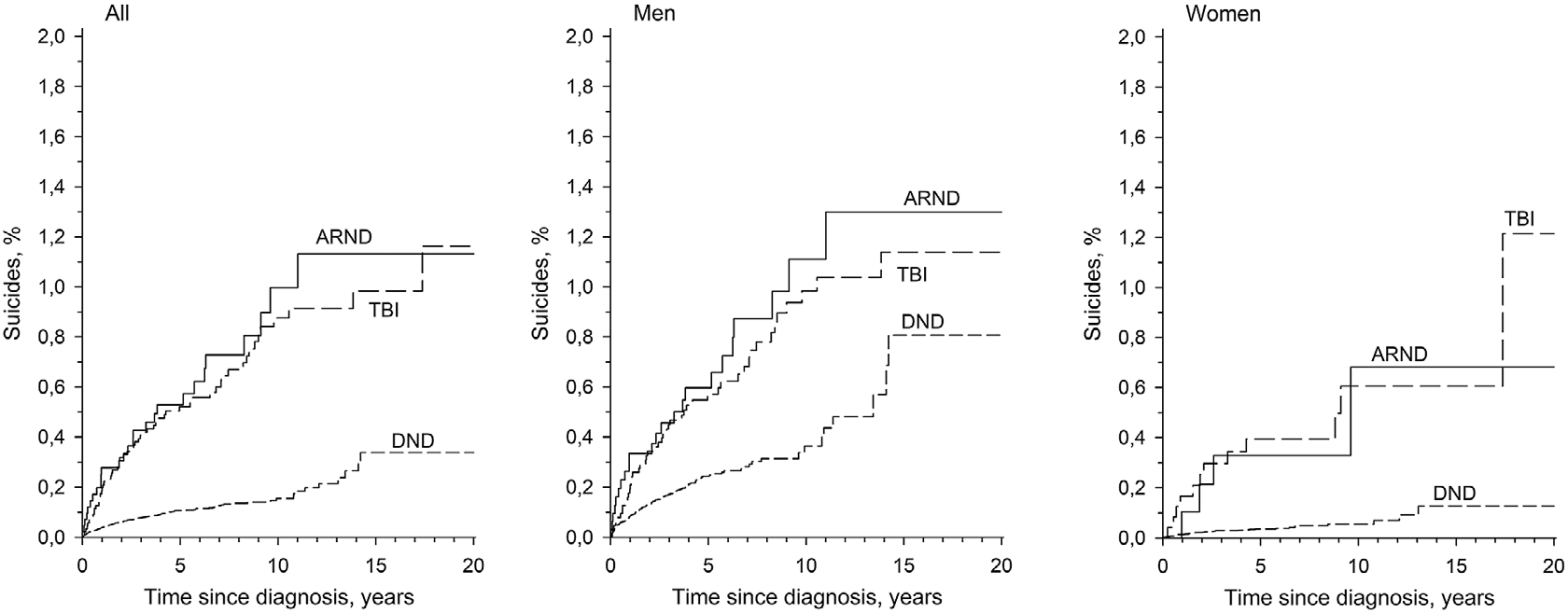
Suicides of patients (Kaplan–Meier estimates) with DNDs, ARNDs, and TBIs in 15 years from diagnosis. Abbreviations: ARNDs, alcohol-related neurocognitive disorders; DNDs, degenerative neurocognitive disorders; TBIs, traumatic brain injuries.

Within the 15-year period, 0.3% (95% CI: 0.2–0.5) of patients with DND, 1.1% (0.7–1.8) of patients with ARND, and 1.0% (0.7–1.3) of patients with TBI died from suicide (Figure 1). The incidence of suicides per 100,000 person-years in the whole research population was 27.8 (24.6–31.3). Men died from suicide more often than women [58.9 (51.3–67.4) versus 9.8 (7.5–12.5) per 100,000 person-years] (IRR: 6.04; 95% CI: 4.54–8.02). Of all three groups of patients, the highest number of suicides per 100,000 was in the ARND group (98.8; 65.1–143.8), followed by the TBI group (82.0; 62.4–105.8) and the DND group (21.2; 18.3–24.5). Suicides per 100,000 person-years annually during the first 10 years from diagnosis were highest in the first year: in the DND group (40.1; 95% CI 32. to 50.1), in the ARND group (279.0; 154.4–503.4), and in the TBI group (185.7; 113.7–303.1).

### Causes of death

The most common cause of death in the whole sample was self-inflicted injury by hanging, strangulation or suffocation, and drowning (*N* = 122), its incidence being 12.4 (10.3–14.8) per 100,000 person-years. The second highest incidence of 5.7 (4.3–7.4) per 100,000 person-years was for self-inflicted poisoning (*N* = 56), and the third highest incidence of 4.7 (3.4–6.2) per 100,000 person-years was for self-inflicted injury by firearms, explosives, smoke, fire, flames, steam, hot vapors, or hot objects (*N* = 46). Differences in methods of intentional self-harm between different patient groups are presented in Table 2.

**Table 2.** External causes of death (intentional self-harm) of patients with DNDs, ARNDs, and TBIs per 100,000 person-years with 95% CI

	DND (*N* = 218,704), incidence (95% CI)	ARND (*N* = 4,248), incidence (95% CI)	TBI (*N* = 8,865), incidence (95% CI)	*p*-Value
X60–X67	3.2 (2.1–4.6)	40.3 (20.1–72.0)	23.6 (13.8–37.8)	<0.001
X70 and X71	10.7 (8.7–62.5)	32.9 (15.1–62.5)	25.0 (14.8–39.8)	<0.001
X72–X78	4.1 (2.8–5.6)	11.0 (2.3–32.1)	9.7 (3.9–20.0)	0.038
X80 and X81	2.8 (1.8–32.1)	11.0 (2.3–32.1)	6.9 (2.3–16.2)	0.025
Y87	0.5 (0.1–1.2)	3.7 (0.1–20.4)	16.7 (8.6–29.1)	<0.001
ALL	21.2 (18.3–24.4)	98.8 (65.1–143)	82.0 (62.4–105)	<0.001

X60–X67: intentional self-poisoning; X70 and X71: intentional self-harm by hanging, strangulation, or suffocation (X70), intentional self-harm by drowning or submersion (X71); X72–X78: intentional self-harm by shooting, explosive material, burning or steaming, or sharp object; X80 and X81: self-harm by jumping from a high place (X80) or jumping or lying before moving object (X81); Y87: sequelae of intentional self-harm, assault, and events of undetermined intent.

Abbreviations: ARNDs, alcohol-related neurocognitive disorders; CI, confidence interval; DNDs, degenerative neurocognitive disorders; N, number; TBIs, traumatic brain injuries.

In detail, patients with ARNDs died more often from hanging or drowning (*N* = 7 + 2), self-poisoning (*N* = 11, of which the group of psychotropics and antiepileptics was *N* = 7), or jumping from a high place (*N* = 3); patients with TBIs from hanging or drowning (*N* = 15 + 3), self-poisoning (*N* = 17, psychotropics and antiepileptics *N* = 9), or late effects of intentional self-harm (*N* = 12); and patients with DNDs from hanging or drowning (*N* = 77 + 18), self-harm by shooting or sharp objects (*N* = 28 + 8), or self-poisoning (*N* = 36, psychotropics and antiepileptics *N* = 16).

### Suicide mortality compared with the general population

Relative to the age- and sex-matched population, SMR for suicides of the total sample was 1.60 (1.42–1.80). It was slightly higher in men (1.63; 1.42–1.86) than in women (1.52; 1.18–1.95).

**Figure 2.**
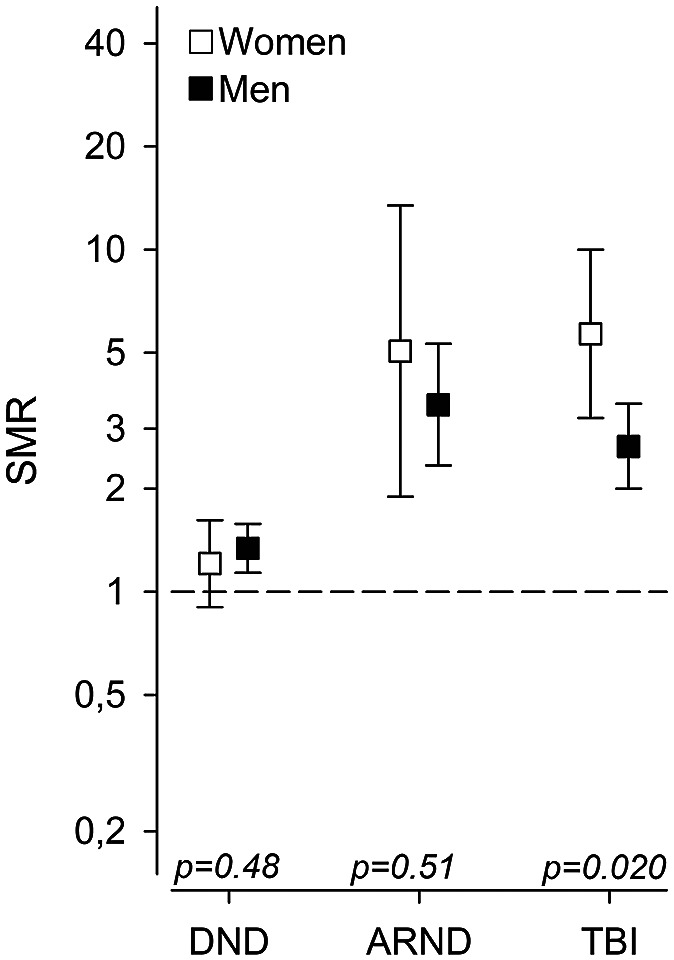
Suicides by sex compared with the same-aged calendar-period-specific (subject-years methods) general population in DNDs, ARNDs, and TBIs. Abbreviations: ARNDs, alcohol-related neurocognitive disorders; CI, confidence interval; DNDs, degenerative neurocognitive disorders; N, number; SMR, standardized mortality ratio; TBIs, traumatic brain injuries.

Mortality ratios for suicides were elevated compared with the general population in all three groups of patients. The highest SMR was found in the ARND group (3.69; 2.53–5.38), followed by the TBI group (2.99; 2.31–3.86) and the DND group (1.31; 1.13–1.51) (Figure 2). When calculating SMRs of groups by sex, it was increased in every patient group, except women with neurocognitive disorder (Table 3).

**Table 3.** SMR for suicides in DNDs, ARNDs, and TBIs by sex

	*N*	SMR
DND	188	1.31 (1.13–1.51)
Women	45	1.21 (0.90–1.62)
Men	143	1.34 (1.14–1.58)
ARND	27	3.69 (2.53–5.38)
Women	4	5.05 (1.90–13.46)
Men	23	3.52 (2.34–5.30)
TBI	59	2.99 (2.31–3.86)
Women	12	5.68 (3.22–10.00)
Men	47	2.66 (2.00–3.55)

Abbreviations: ARNDs, alcohol-related neurocognitive disorders; DNDs, degenerative neurocognitive disorders; N, number; SMR, standardized mortality ratio; TBIs, traumatic brain injuries.

## Discussion

The main finding in our study of patients with DND, ARND, and TBI was that suicide ratios were higher in all three patient groups than in the same-aged general population. Among these groups, the ARND patients exhibited the highest SMR of 4 for suicide. The TBI group had a 3-fold higher SMR, while the DND group showed a significant but comparatively lower SMR of 1.3-fold.

Interestingly, we found that the elevated risk of suicide persisted for more than 10 years after the initial diagnosis in all patient groups, being particularly noteworthy in the DND group. The risk for suicide was most pronounced within the first year of diagnosis. Additionally, we found that men accounted for a greater number of suicides than women. As a cause of suicide, violent methods, such as intentional self-harm by hanging or drowning, were typical, followed by self-poisoning.

Previous studies have reported that older individuals who committed suicide are less likely to have been under the influence of alcohol, to have experienced hospital-treated depressive episodes, or to have had prior physical illnesses than younger individuals (29–32). However, older individuals may exhibit fewer psychiatric symptoms despite having a higher intent to die by suicide (31). Even the presence of passive suicidal thoughts, such as the “wish to die,” may strongly predict premature death [33, 34]. Among those with dementia, there is a decreased likelihood of a history of self-harm, documented psychiatric symptoms, or antidepressant prescriptions before suicide compared with those without dementia (25). Regrettably, the scientific evidence supporting the effectiveness of antidepressants in preventing suicide among older adults remains inconclusive (1).

As far as we know, this comprehensive Finnish nationwide register study is the first to observe the suicide mortality in ARND. It also provides valuable data for comparing the risk of suicide in different CNS–damaged conditions, including DNDs, TBIs, and ARNDs.

### Suicide and DNDs

In this study, which included both inpatient and outpatient settings, the overall incidence of completed suicides among dementia patients was 21.2 per 100,000 person-years. By comparison, suicide mortality among population aged 65 and over in Finland was 18.6 per 100,000 persons in 1998 to 2018 [35]. Our research finding aligns with the incidence rates of completed suicides reported by Schmutte et al. and Erlangsen et al. [9, 25]. Schmutte et al. in their comprehensive study of patients with Alzheimer’s disease and related dementias, reported an incidence of 26.4 per 100,000 person-years [25]. Erlangsen et al. in a longitudinal study of dementia patients from 1980 through 2016, reported an incidence of 57.7 per 100,000 person-years [9]. In our study, the SMR for suicides in the DND group was 1.3, which complements the finding of Schmutte’s 1-year follow-up study where an SMR of 1.5 of was observed [25].

The most critical time for suicidal death in our study, as well as in a South Korean retrospective cohort study [6], was within the first year of dementia diagnosis. Erlangsen et al. reported an overall adjusted IRR of 3 for suicides in dementia patients during the first month after diagnosis, which decreased to less than 0.8 over time [9]. A study of hospital-diagnosed dementia patients noted that the highest risk period for suicide occurred within the first 6 months of diagnosis [7].

In our study, the risk of death by suicide in the DND group stayed elevated for up to 10 years after the diagnosis, a finding in line with the study by Erlangsen et al. [7] with hospitalized DND patients. These results suggest that more advanced cognitive impairment does not necessarily protect individuals from fatal self-harm. A recent systematic review of 30 studies including neurocognitive disorders revealed 29% prevalence of depression, regardless of the type of dementia, and this prevalence remained relatively constant across all stages of cognitive impairment [17]. These findings might partially explain why the risk of suicide persists for a long time. In another systematic review, the suicide risk of AD patients was increased for many years after diagnosis. A particular risk group for suicide were patients who were hopeless and had previous suicide attempts [36]. Consequently, clinicians working with AD patients should appropriately assess each patient’s risk of suicide.

### Suicide and ARNDs

Our study revealed a significant presence of suicidality in ARND. Although we could not find specific literature comparing suicide death rates, our previous study demonstrated an all-cause SMR of 5.4- to 5.6-fold for this patient group [37]. Furthermore, existing literature has consistently shown a high risk of suicide among patients with alcohol use disorders, with an SMR of 8.8 for men and 16.4 for women [38].

The etiology of ARND, including WKS and other alcohol-related cognitive disorders, is believed to be multifactorial, potentially involving the direct neurotoxic effects of ethanol as well as thiamine deficiency [39, 40]. Initially, Wernicke syndrome presents with cerebellar dysfunction, oculomotor symptoms, and changes in mental status. However, 56%–84% of these individuals develop a severe and persistent amnestic disorder known as WKS [40]. In the current study, the highest risk of suicide occurred within the first 12 months of ARND diagnosis, possible due to a heightened awareness of the disease and its consequences compared with later stages of the illness.

Recognizing the elevated suicide risk in ARND patients is crucial, given their relatively young age at diagnosis and the stable or worsening nature of their cognitive symptoms, which predispose them to depression. These patients may require support and surveillance for decades to come.

### Suicide and TBIs

Previous studies support our finding of a three-fold suicide risk in TBI patients compared with the general population [11, 13]. A Danish register conducted by Teasdale examined patients over 40 years old and found SMRs for suicides after TBI ranging from 1.84 to 5.17 depending on the type of TBI and the age subgroup at diagnosis [13]. The highest suicide rate was found in the younger age group [13]. More recently, Madsen et al. published a retrospective register cohort study and reported an SMR of 1.9 in individuals with TBI [11]. Moreover, as in our study, the highest susceptibility to suicidal behavior among TBI patients has been observed shortly after diagnosis [41].

More severe injuries have been associated with greater dysfunction and increased risk of mental health problems and self-harm by self-inflicted means or suicide [10–13, 42]. Notably, in our study, TBI patients were younger, especially compared with DND patients, which may contribute to their relatively better functional capacity to carry out suicide attempts.

### Suicide methods

Violent methods, such as hanging or drowning, were the most common cause of death in all three patient groups. Previous studies consistently have indicated that older generations tend to use more violent and lethal suicide methods than younger generations [4, 24, 43–45].

A 9-year follow-up study of suicidal deaths in demented patients reported that self-poisoning (28%), drowning (19%), and hanging (17%) were the most frequently employed methods [24]. Similarly, in the Northern Finnish cohort of non-demented patients, violent suicide deaths were prevalent, particularly among individuals aged 65 years or older [43]. In the subgroup of individuals aged over 75 years, hanging accounted for 43% of suicides, followed by shooting (17%) and drowning (18%), although data specifically on patients with DND were not investigated separately.

An American study conducted on TBI patients revealed that firearms, especially among military veterans, and poisonings were the most prevalent suicide methods [41]. In our study, firearm suicides were not prominent. However, the choice of suicide method can be influenced by cultural and historical factors as well as by the legislation in a particular country. In Finland, for instance, individuals with moderate neurocognitive disorder or serious impulsive behavior are prohibited from carrying firearms, which may explain the divergence in results.

Patients with ARNDs exhibited self-harm with self-poisoning by medication, especially psychotropics, as the leading cause of death. This pattern may be attributed to the propensity for addiction in this patient group, which should be considered in the treatment of these patients.

### Strengths and limitations

The main strength of our study is the utilization of comprehensive Finnish registers that cover health-care and criminal data for all individuals in both inpatient and outpatient settings, without regard for such factors as age, socioeconomic situation, and place of residence. The sample of DND, ARND, and TBI patients is large, and the follow-up time is sufficiently long to show reliable and qualified findings that earlier studies of these registers also support [30, 46].

As a weakness of our research, we were unable to fully exclude information bias, selection bias, and confounders due to the nature of a register-based study despite the high quality of the registers used. Individual validation of each patient’s diagnosis was not feasible; however, the diagnoses were set following thorough clinical evaluation in accordance with the Finnish Current Care Guidelines [47].

As additional limitations, our study did not include information on alcohol consumption, comorbidities, such social factors as marital status and income level, or the severity of dementia. We lacked data on the extent of neuropsychiatric symptoms associated with these disorders, like depression, or the use of psychotropic drugs. In addition, certain subgroups had relatively small numbers of suicide cases. We also acknowledge that while the results may not be directly applicable to the healthcare systems of other countries, they are nonetheless indicative. We found no previous literature on suicide mortality in ARND patients, but in earlier studies, alcoholic WKS and ARD may have been included under a diagnosis code F10 (alcohol disorder in general).

## Conclusion

Individuals suffering from DNDs or ARNDs, as well as those with sequelae from TBIs, face an elevated risk of suicide compared with the general population. This heightened risk affects both sexes, persists over time, and is likely associated with neuropsychiatric symptoms characteristic of these disorders.

Given the chronic nature of these conditions, it is crucial to maintain ongoing attention to screening of mental health. The risk of suicide appears to be highest in the first year after diagnosis, underscoring the importance of assessing psychiatric symptoms at every appointment. This is especially critical for depression, which is known to be associated with suicidal tendencies. Regular medical and psychological interventions should be implemented to address factors that increase the risk of suicide, given the incurable nature of neurocognitive disorders. We advocate for engaging in daily activities that improve functionality and alleviate feelings of loneliness and helplessness. Preventive measures should focus on reducing excessive alcohol consumption and preventing head injuries, achieved through education and suitable legislation. Furthermore, strategies that aim to mitigate stressors, enhance resilience, promote positive aging, and foster social interactions are recommended.
